# Capacity Building in Remote Facilitation of Newborn Resuscitation

**DOI:** 10.3390/children10061038

**Published:** 2023-06-09

**Authors:** Emily Ahn, Beena D. Kamath-Rayne, Jeffrey Perlman, Sara Berkelhamer

**Affiliations:** 1 Division of Neonatology, New York-Presbyterian Weill Cornell Medicine, New York, NY 10065, USA; ema9066@nyp.org (E.A.); jmp2007@med.cornell.edu (J.P.); 2 Global Newborn and Child Health, American Academy of Pediatrics, Itasca, IL 60143, USA; bkamathrayne@aap.org; 3 Division of Neonatology, Seattle Children’s Hospital, University of Washington, Seattle, WA 98105, USA

**Keywords:** remote training, neonatal resuscitation, simulation, resuscitation education

## Abstract

The past decade has been notable for widespread dissemination of newborn resuscitation training in low-resource settings through simplified training programs including Helping Babies Breathe. Since 2020, implementation efforts have been impacted by restrictions on travel and in-person gatherings with the SARS-CoV-2 pandemic, prompting the development of alternative methods of training. While previous studies have demonstrated feasibility of remote neonatal resuscitation training, this perspective paper covers common barriers identified and key lessons learned developing a cadre of remote facilitators. Challenges of remote facilitation include mastering videoconferencing platforms, establishing personal connections, and providing effective oversight of skills practice. Training sessions can be used to support facilitators in acquiring comfort and competency in harnessing videoconferencing platforms for effective facilitation. Optimization of approaches and investment in capacity building of remote facilitators are imperative for effective implementation of remote neonatal resuscitation training.

## 1. Introduction

An estimated 2.3 million newborns die globally each year, with the greatest burden of mortality occurring in low-resource settings [1]. Perinatal asphyxia (or an intrapartum-related event) is the leading cause of neonatal mortality only behind prematurity [2]. Quality resuscitative care has the potential to prevent many of these deaths, and to reduce the burden of long-term developmental morbidities associated with perinatal asphyxia. Training in basic or simplified newborn resuscitation, as provided using the Helping Babies Breathe (HBB) program, has been shown to improve outcomes in a range of clinical environments, with meta-analyses identifying both decreased rates of stillbirth and a 30% reduction in first day neonatal mortality [3,4,5].

In the low-resource setting, HBB has expanded access to resuscitation training by providing a low-cost, simplified, pictorial curriculum that is easy to understand at all learning levels. The program is designed for small group, interactive facilitation with use of hard copy teaching materials and in-person skills practice sessions using a portable manikin. In-person facilitation allows for tailored sessions and local adaptation by incorporating participants’ experiences and discussion of site-specific needs. Additionally, facilitators are able to give hands on feedback during practice sessions ensuring each participant can adequately perform resuscitation skills. The program has been widely disseminated over the past decade, including training of over 850,000 birth attendants in 80 countries and translation into over 27 different languages [6].

However, in-person training of HBB was either halted or limited in many locations during the SARS-CoV-2 pandemic. This prompted exploration of alternative ways to deliver neonatal resuscitation training, including remote presentation of training content via teleconferencing platforms. Early remote training sessions utilized an animated PowerPoint of the next version of HBB that was already in development with the World Health Organization, entitled Essential Newborn Care. This approach lacked much of the interaction typically present with HBB sessions. In an effort to continue its emphasis on techniques to engage adult learners and provide immediate access to the training program, the American Academy of Pediatrics partnered with Laerdal Global Health in developing an online platform, ENC Now! (www.hmbs.org). The interactive design of this online program incorporates videos, demonstrations, assessments, and the option to utilize a flipped classroom model with a video avatar. Skills are reviewed with a remote facilitator utilizing videoconferencing.

Although the challenges to continuing in-person training were not unique to HBB, the program’s piloting of remote facilitation has highlighted key lessons in transitioning in-person facilitators to a remote format. This perspective paper covers lessons learned while transitioning to remote facilitation of HBB as well as the tools implemented through facilitator training sessions to empower individuals in their transition from leading in-person to leading remotely. The lessons learned and tools developed have future application beyond training within a pandemic as remote facilitation presents an opportunity to expand the influence and support further dissemination of newborn resuscitation training. Remote facilitation lessens barriers such as travel time for facilitators and participants and the associated expenses [7,8]. It can also increase intra- and international collaborations [8] and be used to reach areas that do not have the personnel to support training efforts. Lastly, training remotely facilitates integration of complementary educational materials including clinical videos or other online content. However, in order to ensure quality programming and implementation, capacity building of remote facilitators must be addressed.

## 2. Feasibility of Remote Teaching

While global estimates for rates of use of remote training are unknown, a scoping review of telesimulation use in healthcare education published in 2023 found 29 articles across multiple disciplines including adult, obstetric, pediatric, and neonatal scenarios and confirmed recent growth of this approach. Of the 29 articles, 62% of the programs were developed in response to the SARS-CoV-2 pandemic. However, all but two of the studies were conducted in high resource areas and the feasibility and application in lower resource environments remains less clear [9].

Despite being highly hands-on and skills-based, remote training of neonatal resuscitation has been shown to be both feasible and effective. Published evaluations from well-resourced settings have characterized successful training. A study of 18 medical students and neonatal nurses in Austria found the Neonatal Resuscitation Program (NRP) algorithm and resuscitation skills could be taught via telesimulation utilizing one-hour classes and small classroom sizes of two to three participants. Pre/post knowledge test scores improved significantly and participants indicated they enjoyed the sessions; however, they were critical of the quality of instructor feedback and supervision [10]. Similarly, a study of 14 previously NRP-trained but HBB-naive residents at the University of Massachusetts evaluated remote as compared to in-person HBB training. Remote learners were gathered in one conference room while videoconferencing with the facilitator. A survey assessing participant’s confidence with the content of the program and skills performance was utilized before and after the course. The confidence of learners significantly increased with both training approaches and did not differ between groups, suggesting that remote training is similarly effective in improving confidence of learners with a neonatal resuscitation background [11].

These results have been replicated with other neonatal programs. In Manitoba, Canada, videoconferencing versus face–face delivery of S.T.A.B.L.E.^®^ (the Sugar, Temperature, Airway, Blood pressure, Lab work, and Emotional support program for neonatal stabilization), was evaluated amongst 56 multidisciplinary learners. Pre/post knowledge assessment demonstrated a significant increase in both groups and no difference between the two approaches [12]. A second study by the same investigators determined that an experienced remote instructor can accurately assess the practical skills of a provider, including bag-mask ventilation, during a simulated neonatal code over real-time videoconferencing. Comparisons of remote and in-person instructor observations of 18 simulated codes demonstrated 2.8% inter-observer variability for quantitative data and consistent written observations of performance [8].

These findings are substantiated by a broader scoping review of remote simulation-based training that included pediatric and adult resuscitation [13]. Nine non-neonatal studies were identified with remote training and simulation in emergency care, pediatric resuscitation, cardiopulmonary instability, and respiratory failure. Variable outcomes were assessed. Four studies looked at technical feasibility; three demonstrated telesimulation was technically feasible while one found that communication was a barrier to understanding the case. Three studies specifically assessed learning outcomes; two showed an improvement in performance when looking at remote facilitation and one found no significant difference between test scores in those who received in-person versus remote simulation. The one study that assessed facilitator perceptions reported difficulty in assessing participant’s emotions and facial expressions [13].

While these studies collectively support remote training, only a limited number of studies focused on neonatal resuscitation in low-resource settings have been conducted. Effective learning of HBB has been demonstrated with a hybrid model involving a remote facilitator to present content paired with an in-person skills trainer. With this model, Guatemalan nurses and nursing students demonstrated an average increase in their pre/post knowledge tests by 12% with all participants passing the skills assessment [7]. A study conducted in Mindanao, Philippines, with nursing students explored skill decay, utilizing in-person versus video-based refresher training of HBB after first receiving an in-person HBB course. The study found no significant difference between the two groups for time to bag mask ventilation and performance on knowledge and practical examinations two months after having received a refresher course [14].

A fully remote study performed in Delhi, India, randomized 48 in-service staff nurses to receive NRP-based lectures and demonstrations in a classroom or via tele-education instruction [15]. Knowledge and skills attainment were assessed through in-house discussion and mock resuscitations. Both groups demonstrated a significant increase in knowledge and skills scores; however, knowledge scores were statistically higher following classroom training after adjusting for the difference in pre-training baseline scores. Satisfaction with content and evaluation of personal gain were similar in both groups but satisfaction with active participation and presentation of the content was scored higher in the classroom as compared to the remote group [15]. Collectively, these studies demonstrate the feasibility of remote facilitation of newborn care while highlighting the need for strategic implementation given the diverse learner responses.

## 3. Challenges of Remote Facilitation

Our experience orienting a range of international facilitators from low-resource and resource replete settings to remote training has identified numerous challenges. The most notable challenge observed was facilitators’ discomfort with use of remote platforms and new technology. While many have exposure to videoconferencing as a participant, the unique setup, layout, and features of the numerous platforms (i.e., Zoom, Microsoft Teams, Skype, Cisco Webex) leave many people intimidated to attempt remote facilitation. As a participant on these platforms, one plays a passive role with use of limited tasks such as muting or unmuting and commenting in chats. Many people have experience limited to one platform that is common in their institution. A misperception in taking on remote facilitation is that mastery of every platform is necessary and knowledge of numerous advanced tasks is needed. This can be daunting as while platforms may be similar in function, they possess unique layouts and features. For example, Zoom’s chat is located on the bottom of the screen on a permanent menu bar whereas Microsoft Teams has a meeting chat at the top of the screen and a separate application chat on the left of the screen that contains history of chats separate from the meeting. These nuances can be a barrier to attempting remote facilitation.

Similarly, an inability to effectively harness interactive videoconferencing tools, such as annotations, reactions, or polling, results in many skilled trainers defaulting to videoconferencing basics such as screen-sharing a PowerPoint. This can lead to lecture-based teaching rather than interactive facilitation, potentially impacting the efficacy of participant learning. A lack of familiarity with videoconferencing platforms can waste valuable course time for setup and create a distraction for many participants.

Another challenge involves barriers in establishing personal connections. Videoconferencing reduces social presence, the subjective feeling of being present with a real person [16]. Social presence can be important in cultivating relationships and emotions that enhance the learning experience [13]. Integral to social presence is behavioral feedback that is inherently provided in face–face communication such as eye contact, head nodding, verbal cues, and facial expressions. This feedback can indicate levels of attentiveness and comprehension of content [16]. During videoconferencing, participants are often muted and not visible to the facilitator due to screen-sharing or cameras being turned off to reduce the data burden. This leaves the facilitator disconnected and impacts efforts for interactive teaching. Moreover, maintaining both participant and facilitator engagement can be a challenge. Videoconferencing inherently creates an environment filled with distractions as participants have constant access to the internet. They may be participating from an individual screen, reducing the peer pressure to actively participate that comes from being in a group or classroom setting.

The HBB and ENC curricula are structured around skills practice sessions. In-person master trainers are adept at transferring neonatal resuscitation skills to participants through one-on-one hands-on practice. With videoconferencing, those resuscitation skills are transferred to the participant through demonstrations and verbal feedback. To be a strong remote facilitator, one has to optimize their camera skills and enhance verbal feedback and direction. This can be difficult when the participant skills practice is performed with one camera as a large group instead of individual practice sessions. Lastly, the benefits of remote training such as reducing travel and establishing new collaborations brings a novel set of challenges. Differences in time zones require coordination of schedules and can make arranging lengthy sessions difficult.

**Figure 1:**
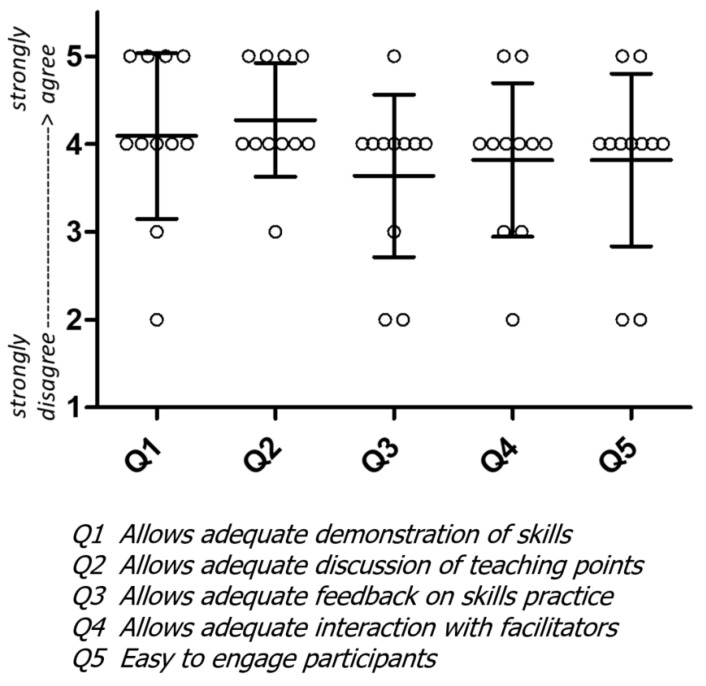
Facilitator perception of remote training. Feedback from facilitators utilizing various beta versions of ENC Now! for remote training in 11 different countries.

An informal survey of experienced HBB facilitators’ perceptions of remote training in newborn resuscitation was performed in 2021 and supported many of these concerns [17]. There was less agreement with statements that remote training allowed adequate feedback, engagement, and interaction with facilitators (Figure 1), highlighting the need for proactively addressing the gaps, building expertise in remote training, and incorporating novel approaches to optimize interactions.

## 4. Overcoming Challenges in Creating Remote Facilitators

### 4.1. Establishing Videoconferencing Skills

Our experience orienting a range of international facilitators to remote training also highlighted opportunities for building competency in remote facilitation. Fear and discomfort with technology were mitigated with virtual remote facilitator training sessions designed for and adapted to participants’ needs. These sessions focused on the most common videoconferencing platform that participants planned on utilizing. Mastering one platform was felt to be more manageable and impactful than becoming facile in multiple. Imperative to achieving competence in a platform is learning its interface on various devices. The design and functions of an individual platform often vary between mobile devices, tablets, and computers, all of which may be used by participants. In addition, institutional platform accounts can have pre-selected settings that may limit or modify certain functions, such as reactions or chat boxes, impacting use. Therefore, new remote facilitators were encouraged to practice using videoconferencing on the institutional account they planned on utilizing for remote HBB training.

Remote facilitation training sessions can be used to demystify the complexities of remote teaching for facilitators who might have a range of technology backgrounds. Step-by-step demonstrations, slideshow instructions, and review videos of platform tools can start with very basic steps to create a solid foundation without assuming background knowledge. We found that many facilitators were familiar with being a participant of videoconferencing but had limited experience beyond attendance. Therefore, while the terminology of tasks such as sharing a screen were familiar, the practicalities of how the platform changes when completing that task were often foreign. For example, when sharing a screen, some platforms will automatically change where and how many participant videos are seen on the facilitator’s screen. This unfamiliarity with presenter views and how to manipulate them were addressed during the facilitator training practice sessions.

**Figure 2:**
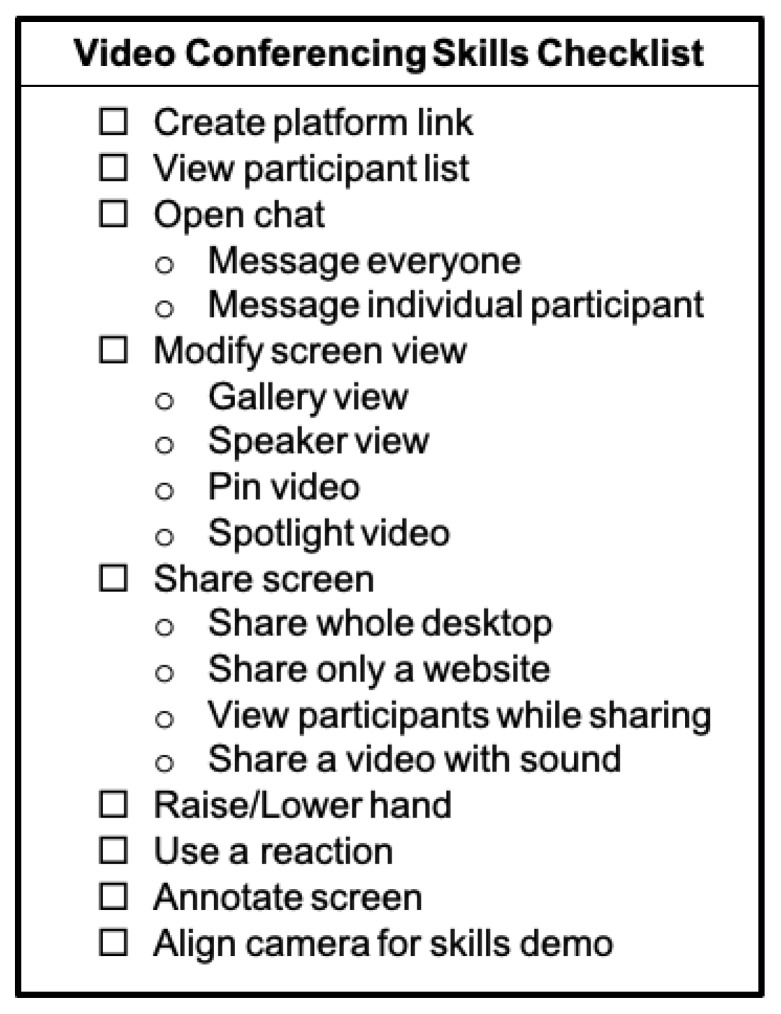
Checklist of common videoconferencing functions for remote facilitators to master.

Practicing teaching online is a powerful tool for remote facilitator capacity building. When possible, orientations involved small breakout sessions where session leaders were paired with less experienced remote facilitators. The goal of these practice sessions was to gain familiarity and provide mentored experience with the videoconferencing platform. Participants practiced basic functions including sharing a screen, sharing video sound, pinning or spotlighting a participant screen, playing a video recording, and manipulating the facilitator screen with guided support by session leaders. A check list of common features a remote facilitator should be familiar with to achieve comfort can be used for independent or group learning (Figure 2). These features are nearly universal in videoconferencing platforms. Demonstrations of many of these functions can be found on each platform’s help page.

### 4.2. Increasing Participant Engagement

**Figure 3:**
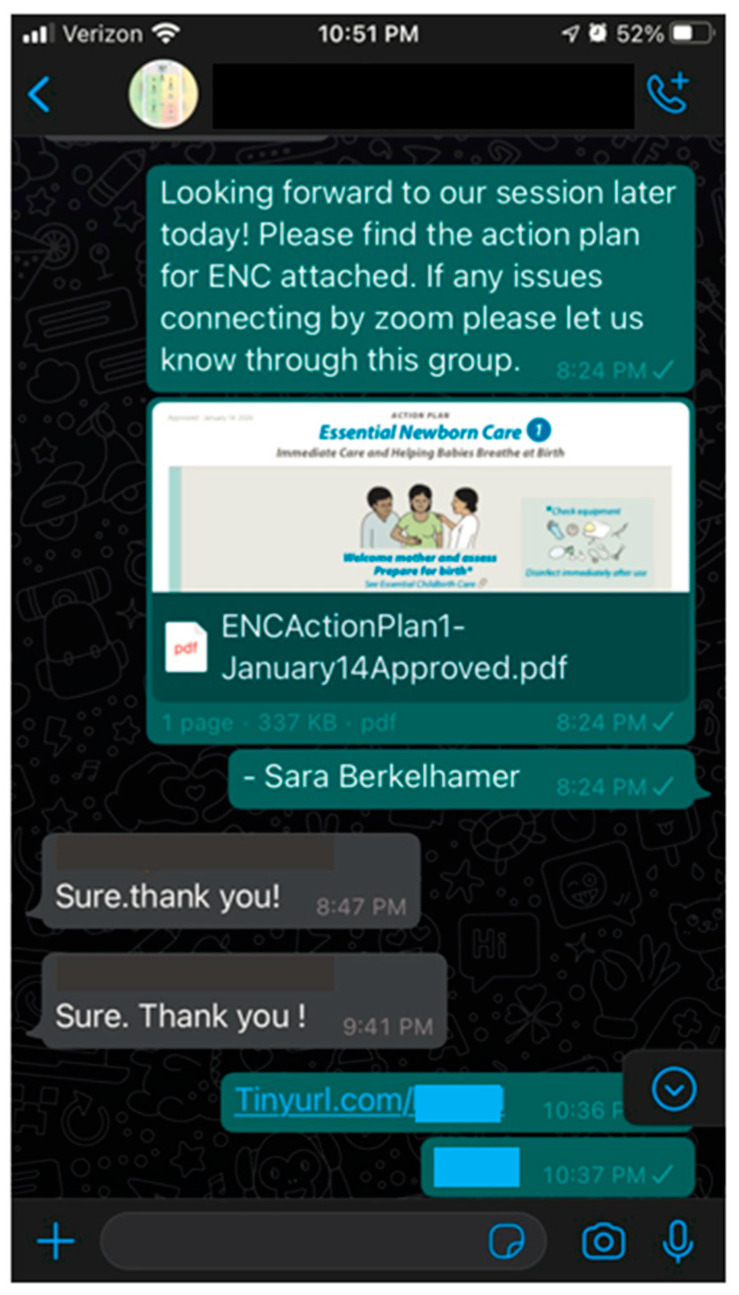
Sample WhatsApp group chat. Prior to remote facilitation of ENC Now!, the facilitator created a group chat for sharing of material and maintaining communication.

Once baseline videoconferencing skills are established, the facilitator training sessions can focus on strategies to increase personal connections and participant engagement over videoconferencing. If participant lists are available prior to training, a group chat can be established over a common messaging application used in the participants’ country (i.e., WhatsApp, Telegram, or WeChat). This group chat provides a way to share materials directly with the participants and increases individual participant access to the facilitator (Figure 3). If videoconferencing becomes disconnected during a course, communication can be reestablished or continue through the group chat.

Multiple strategies can be utilized with remote training to increase participant engagement, many of which do not require advanced technology. Many platforms allow participants to change their name visible to other participants that are in the session. Facilitators can encourage participants to place their preferred name, professional background, and/or work location in that space to increase familiarity among participants. Scheduling increased time for introductions and ice breakers that involve all participants encourages connections and participation. For classrooms where participants are soft spoken or in areas where participant video is unavailable due to internet bandwidth or data use concerns, incorporating polls can increase participation. Polling software may be unfamiliar for many, so utilizing existing platform functions such as reactions, including thumbs up/thumbs down, or the chat box can increase engagement without overwhelming participants. Similarly, given the challenges of interpreting facial feedback, the facilitator may need to be more direct in assessing understanding by asking content questions and pausing for participant questions. Facilitators should wait a longer period then natural for a response from participants due to delays with unmuting the microphone. Mastering the skills of provider participation while videoconferencing is imperative to maintaining the goal of interactive, engaged group learning.

### 4.3. Classroom Design

The classroom design for remote training can help limit distractions that accompany videoconferencing. Sobelman’s study compared in-person versus remote teaching of HBB and found that one large screen instead of individual workstations supported group learning [11]. Shared screens can limit distractions and foster team learning. Additionally, encouraging participation in a quiet room can reduce interruptions from coworkers or family members and help ensure the facilitator is audible. However, when utilizing one screen for a group of participants, individualized platform functions, such as reactions, can no longer be used. It may also become difficult for the facilitator to see all the participants and hear questions. Requesting that the group assign a spokesperson can encourage interaction and ensure questions from participants are not missed. When utilizing this single screen model, additional portable screens may be helpful to oversee individual skill sessions.

### 4.4. Mastering Skills Demonstration

**Figure 4:**
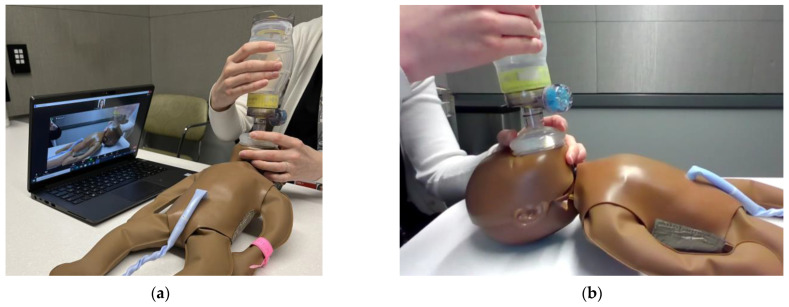
Optimal camera set-up during remote bag-mask ventilation demonstrations. (**a**) Facilitator set-up with camera angled perpendicular to the long axis of manikin; (**b**) Participant view on videoconferencing platform.

Remote facilitation creates new challenges with demonstration of skills over video conferencing. Key to successful visual communication is optimization of camera angle. Cronin’s study on the efficacy of videoconferencing to assess neonatal resuscitation skills found that positioning the manikin with the long axis perpendicular to the camera view was optimal to confirm chest movement during bag-mask ventilation [8] (Figure 4). An adjustable camera such as a phone, laptop, or detachable webcam, allows for multiple views to be obtained and the ability to angle the camera towards the manikin and demonstrator’s hands. Loewen found enlisting local coordinators ensured the camera was focused correctly on the demonstration [12].

**Figure 5:**
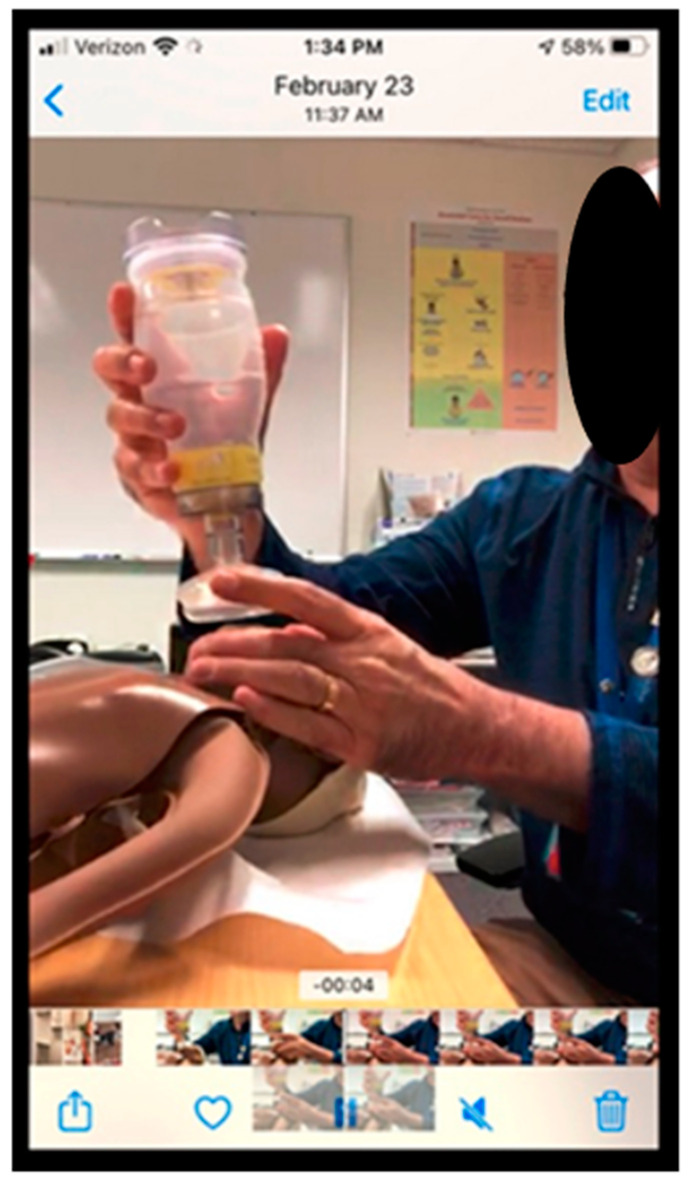
Self-captured bag-mask video demonstration shared via WhatsApp.

Another key to successful skills demonstration is optimizing videoconference optics. Ideally the person demonstrating the skills should be the largest image on the screen. To achieve this, screen-sharing should be discontinued for demonstrations. Some platforms allow you to select one video (“spotlight”) to be the dominant screen for all participants regardless of whom is talking. This is helpful for when the remote facilitator is demonstrating. When the facilitator is monitoring multiple participants practicing skills at once, the facilitator can enlarge one person’s video (“pin”) to ensure best visualization without affecting the screen of other participants. If sufficient facilitators are present and internet bandwidth allows, breakout rooms can optimize the facilitator– participant ratio allowing for more personalized feedback. When bandwidth is limited or hindering video clarity, participants can video record skills practice sessions and send them back to the facilitator through the group messaging applications mentioned previously (Figure 5). This does not allow for real-time feedback but does ensure the monitoring of skills. Increased time for skills practice should be built into the course agenda to allow participants to correct their mistakes and send new recorded videos. Alternatively, when connectivity or optimal observation is a limiting factor, onsite skills facilitators should be considered. These are often individuals who have mastered the skills of neonatal resuscitation but may not have the experience to administer a neonatal resuscitation course. They can also provide insight in to local practices.

### 4.5. Overcoming Language Barriers

As videoconferencing can involve distant collaborations, language barriers may arise. Some videoconferencing platforms allow for real-time closed captioning which can increase comprehension for those participating in their non-primary language. Facilitators should explore language barriers prior to their session and be attentive to slowing down their pace of speaking and building in room for comprehension checks. Despite this, on-site translators may be necessary.

### 4.6. Pre-Course Planning

Practice is essential to complete the transition from becoming an in-person to a remote facilitator. Developing an outline and rehearsing transitions can improve facilitator delivery [8]. Pre-course planning can reduce technical delays due to audio, poor camera positioning, or connectivity issues. Naturally, remote facilitation is associated with longer course duration which should be factored in pre-course planning. Remote facilitation requires a new skill set that can be mastered with mentorship and practice, allowing the facilitator to best focus on the learners instead of the logistics of the process.

## 5. Conclusions

Over the past few years, videoconferencing has become an essential part of learning in many environments. The worldwide adoption of the technology and the economical, temporal, and collaborative benefits create prime opportunities for it to be used globally in neonatal resuscitation training. It is imperative that time is invested into training and supporting remote facilitators for implementation of videoconferencing to be successful. Gaps in the literature remain regarding whether remote facilitation can result in adequate behavioral change and improved clinical care outcomes, however, this question can only be addressed following adequate attention to the quality and optimal delivery of remote training. This perspective piece provides guidance on assisting experienced facilitators with transitioning to remote formats, however, formal evaluation of orientation or training approaches is needed to understand impacts on facilitator skill and comfort in remote facilitation.

## Data Availability

The data presented in this article are available in Figure 1.
